# Signature of Circulating MicroRNAs as Potential Biomarkers in Vulnerable Coronary Artery Disease

**DOI:** 10.1371/journal.pone.0080738

**Published:** 2013-12-05

**Authors:** Jingyi Ren, Jing Zhang, Ning Xu, Guanping Han, Qiang Geng, Junxian Song, Sufang Li, Jianqing Zhao, Hong Chen

**Affiliations:** 1 Department of Cardiology, Peking University People's Hospital, Beijing, China; 2 Department of Medicine, Karolinska Institutet, Stockholm, Sweden; 3 National Engineering Research Center for Beijing Biochip Technology, Beijing, Chidoca; University of Udine, Italy

## Abstract

**Aims:**

MicroRNAs (miRNAs) play important roles in the pathogenesis of cardiovascular diseases. Circulating miRNAs were recently identified as biomarkers for various physiological and pathological conditions. In this study, we aimed to identify the circulating miRNA fingerprint of vulnerable coronary artery disease (CAD) and explore its potential as a novel biomarker for this disease.

**Methods and Results:**

The Taqman low-density miRNA array and coexpression network analyses were used to identify distinct miRNA expression profiles in the plasma of patients with typical unstable angina (UA) and angiographically documented CAD (UA group, *n* = 13) compared to individuals with non-cardiac chest pain (control group, *n* = 13). Significantly elevated expression levels of miR-106b/25 cluster, miR-17/92a cluster, miR-21/590-5p family, miR-126*, and miR-451 were observed in UA patients compared to controls. These findings were validated by real-time PCR in another 45 UA patients, 31 stable angina patients, and 37 controls. In addition, miR-106b, miR-25, miR-92a, miR-21, miR-590-5p, miR-126* and miR-451 were upregulated in microparticles (MPs) isolated from the plasma of UA patients (*n* = 5) compared to controls (*n* = 5). Using flow cytometry and immunolabeling, we further found that Annexin V^+^ MPs were increased in the plasma samples of UA patients compared to controls, and the majority of the increased MPs in plasma were shown to be Annexin V^+^ CD31^+^ MPs. The findings suggest that Annexin V^+^ CD31^+^ MPs may contribute to the elevated expression of the selected miRNAs in the circulation of patients with vulnerable CAD.

**Conclusion:**

The circulating miRNA signature, consisting of the miR-106b/25 cluster, miR-17/92a cluster, miR-21/590-5p family, miR-126* and miR-451, may be used as a novel biomarker for vulnerable CAD.

**Trial Registration:**

Chinese Clinical Trial Register, ChiCTR-OCH-12002349.

## Introduction

Angiographic and pathological studies have shown that most cardiac events, such as myocardial infarction (MI) and sudden cardiac death, are related to rupture-prone, or “vulnerable” plaques. Vulnerability is considered to be the most important factor for the clinical consequence of a plaque [1]. Although several imaging techniques and biomarkers have been developed to characterize vulnerable plaques, these attempts remain critically insufficient [2]. Plaques with similar characteristics often have different clinical presentations. Moreover, factors other than the plaque itself, such as blood coagulability, inflammatory processes, endothelial dysfunction, and the electrical instability of the myocardium, also affect the clinical outcome [3]. As our understanding of coronary artery disease (CAD) has evolved from a focal to a systemic disease, systemic approaches to identify vulnerable patients have become preferable to the identification of local vulnerable plaques or myocardial damage.

MicroRNAs (miRNAs) are ∼22-nucleotide-long noncoding RNAs that regulate the expression of target genes by binding to their 3′ untranslated regions [4]. To date, over 1900 miRNAs have been identified in human, which are predicted to regulate ∼60% of all protein-coding genes [5]. In the cardiovascular system, miRNAs have been shown to play crucial roles in pathophysiological conditions, such as endothelial dysfunction, inflammation, apoptosis, and angiogenesis [6], [7]. Interestingly, miRNAs exist in the circulation in very stable forms [8]. They are packed in transport particles, such as microparticles (MPs), exosomes, and protein complexes, which protect the miRNAs from degradation [9]. Among these particles, MPs were reported to be the major carriers of miRNAs in the blood [9], [10].

Recent studies have reported that circulating miRNA levels may reflect pathological conditions, and some circulating miRNAs have been identified as novel biomarkers with the potential to improve disease diagnosis in clinical practice [8], [11], [12]. However, there is limited information on the value of circulating miRNAs as biomarkers to identify high-risk unstable CAD patients, despite the urgent need for early diagnosis to prevent myocardial damage. Thus, this study aimed to identify the circulating miRNA signature in vulnerable CAD and explore its potential as a novel biomarker for this disease.

## Methods

The protocol for this trial and supporting CONSORT checklist are available as supporting information; see Checklist S1 and Protocol S1.

### Ethics statement

The protocols were approved by the ethics review board of Peking University People's Hospital (Clinical Trials Register No. 2011-92, Beijing, China) and were registered in the Chinese Clinical Trial Register (registration number: ChiCTR-OCH-12002349, registry URL: http://www.chictr.org/en/proj/show.aspx?proj=3153). Informed written consent was obtained from each participant.

### Study population

**Figure 1:**
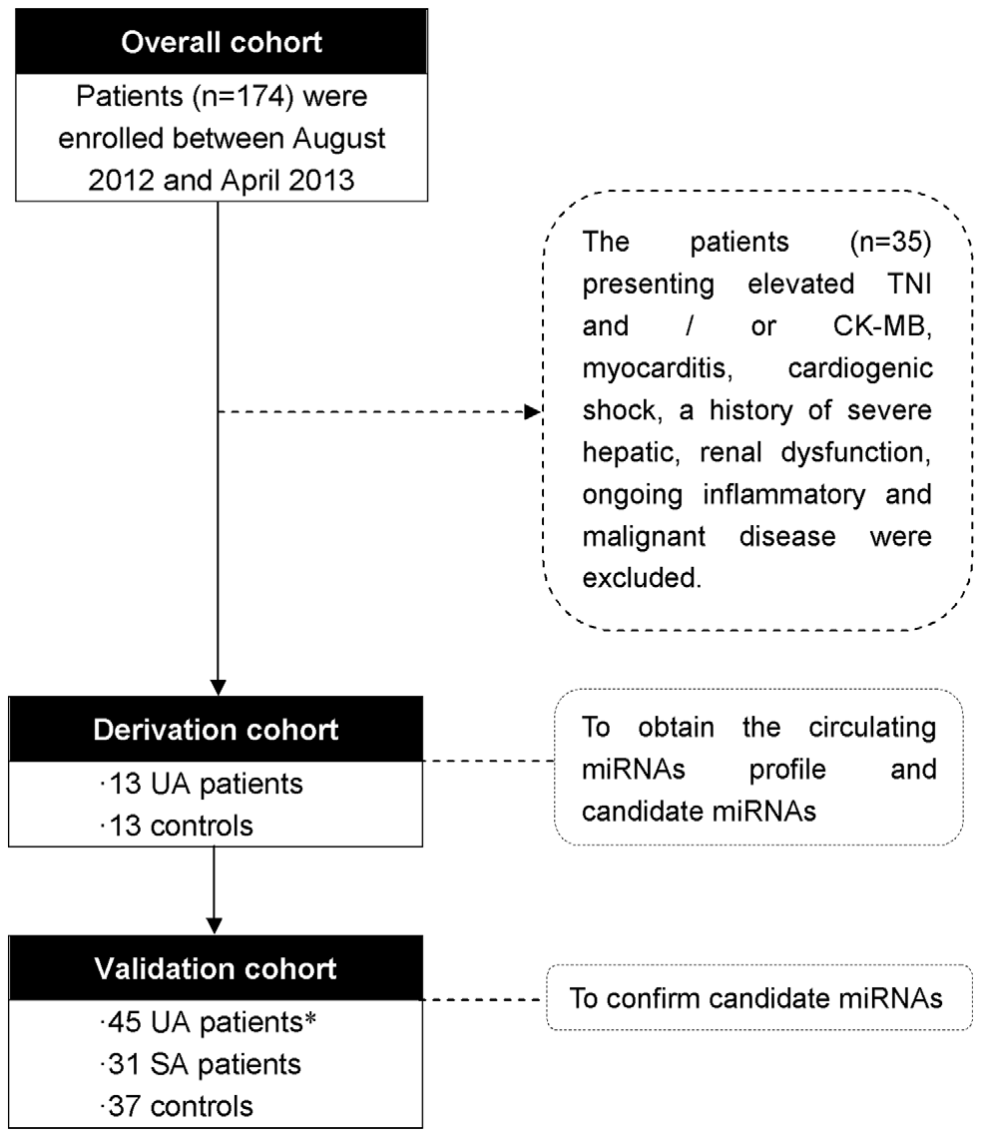
Study flow diagram. All patients were enrolled at Peking University People's Hospital between August 1, 2012 and April 18, 2013. UA, unstable angina; SA, stable angina. * 10 patients in UA group underwent intravascular ultrasound (IVUS) confirming plaque rupture.

All patients were enrolled at Peking University People's Hospital between August 1, 2012 and April 18, 2013. The schematic diagram of this study is summarized in Figure 1. The derivation cohort included two groups that were classified according to angiographic evidence and clinical evaluation of chest pain. Patients with chest pain or discomfort but with angiographic exclusion of coronary atherosclerosis were enrolled in the control group (*n* = 13). Chest discomfort referred to the following complaints: chest pain, pressure, tightness, or heaviness; pain that radiated to the neck, jaw, shoulders, back, or one or both arms; and persistent shortness of breath. Patients with typical unstable angina (UA) and angiographically documented CAD were enrolled in the UA group (*n* = 13).

The results obtained in the derivation cohort were further analyzed in a validation cohort of 37 controls with non-cardiac chest pain, 31 patients with stable angina (SA), and 45 UA patients. Plaque rupture was confirmed in 10 of the 45 UA patients by intravascular ultrasound (IVUS). A ruptured plaque was defined as containing a cavity that communicated with the lumen with an overlying residual fibrous cap fragment. Diagnoses of UA and SA were made according to the ACC/AHA 2007 guidelines for the management of patients with UA/non–ST-elevation MI and the ACC/AHA/ACP-ASIM 1999 guidelines for the management of patients with chronic SA. All UA patients presented with new transient ST-segment abnormalities (≥0.05 mV) that developed during a symptomatic episode and resolved when the symptoms had resolved. All patients in the control group presented with normal ECG and had no evidence of ischemia during exercise ECG. The exclusion criteria were as follows: (1) UA caused by other etiological mechanisms (*e.g.*, coronary focal spasm, coronary artery dissection); (2) secondary UA related to precipitating factors, such as anemia, fever, tachycardia, hypotension, etc.; (3) post-MI UA; (4) elevated troponin I (TNI) or creatine kinase (CK-MB) levels; (4) history of severe hepatic and renal dysfunction; and (5) leukemia, leukopenia, thrombocytopenia, or ongoing inflammatory and malignant diseases.

### Plasma samples collection

EDTA-blood samples were obtained before the cardiac catheterization procedure and were processed within 30 min of collection using two-step centrifugation. Samples were first centrifuged at 1.200 g for 10′ at 4°C. The supernatant were collected and then centrifuged again at 12.000 g for 15′ at 4°C. The supernatant was transferred to RNase-free tubes and then stored at −80°C.

### Extraction RNA from plasma

RNA was isolated from plasma using miRNeasy Mini Kit (Qiagen, Valencia, CA). Briefly, 250 µl of EDTA-plasma were mixed with 700 µl Qiazol, incubated for 5 min at room temperature and subsequently mixed with 140 µl chloroform. The organic and aqueous phase was separated by centrifugation at 12,000 g for 15 min. The upper aqueous phase was collected and the RNA was precipitated by adding 100% ethanol. The mixture was applied to a miRNeasy Mini spin column. After washed several times, the RNA was eluted in 25 µl RNase-free water.

### Taqman low-density miRNA array (TLDA)

A total of 15 ng of total RNAs were reverse-transcribed with Taqman miRNA reverse transcription kit and Taqman miRNA Multiplex RT assays (human pool). The reverse transcription products were then preamplified with Taqman PreAmp kit (Applied Biosystems, Foster City, CA, USA). The preamplification reaction products were analyzed with Human MicroRNA TLDA card A+B version 3.0 (Applied Biosystems), which can detect 754 mature miRNAs simultaneously. The microRNA profiling data were deposited in the public database Gene Expression Omnibus (GEO) (https://www.ncbi.nlm.nih.gov/geo/) with accession number GSE49823.

All steps were performed using a 7900HT Fast Real-Time PCR System. Results were expressed as Cts. The miRNAs with Ct <40 in at least 21 out of 26 samples were considered to be expressed. Raw data were analyzed using Data Assist software for TaqMan gene expression assays version 2.0 (Applied Biosystems). The miRNA expression was normalized to the U6 expression level, which remained constant in all of the samples across the different patient groups. To identify miRNAs that were consistently differentially expressed, the data were subjected to significance analysis of microarrays (SAM). The miRNAs that showed at least 8-fold change and a q-value <0.0001% were considered to be differentially expressed.

### Real-time reverse-transcription polymerase chain reaction (RT-PCR)

The miRNAs were analyzed by real-time RT-PCR using TaqMan miRNA reverse transcription and miRNA assay kits (Applied Biosystems), according to the manufacturer's instructions. Preamplification steps were performed with the preamplification mix and Megaplex PreAmp primers human pool A and B (Applied Biosystems). The target miRNA expression was normalized between different samples based on the U6 expression level and expressed as 2^−(CT[miRNA]-CT[U6])^. The expression data of target miRNAs are presented in logarithmic form.

### Isolation and characterization of MPs from plasma samples

MPs were isolated from human plasma samples as described previously [13]. MPs were analyzed on a FACS Aira flow cytometer (Beckton Dickinson, BD). A known amount of TruCount calibration beads (Beckton Dickinson, BD) was added to each sample before flow cytometry analysis. MP concentration was assessed by comparison to TruCount beads [13]. MPs gate was defined as particles from 0.1 to 1 μm in size using calibration beads (Beckton Dickinson, BD). Presence of phosphatidylserine at the surface of plasma MPs was assessed using fluoroisothiocyanate-conjugated Annexin V [13]. To further identify the cell origin of plasma MPs, MPs were stained with anti-CD31-phycoerythrin.

### Extraction and detection of miRNAs from plasma MPs

Total RNAs were extracted from the isolated MPs using miRNeasy Mini Kit (Qiagen) as described previously [10]. Fifteen ng RNA was used to perform Real-time PCR analysis. Target miRNA expression value was normalized between different samples based on the value of hsa-miR-24, which is one of the most stably expressed references across samples, and are expressed as 2^−(CT[miRNA]-CT[hsa-miR-24])^. The relative expression level of target miRNA in MPs was further adjusted to MP counts.

### Statistical analyses

Quantitative data are presented as the mean ± standard deviation (SD) or standard error of the mean (SEM) for normally distributed continuous variables, or as the median for non-normally distributed continuous variables. For continuous variables, Student's *t*-test or the Mann-Whitney *U* test was used, as appropriate, for group-wise comparisons. For categorical variables, the *χ^2^* test or Fisher's exact test was used. All tests were 2-sided. A significance level of *P*<0.05 was considered to be statistically significant. SPSS 13.0 was used for all statistical analyses.

## Results

### Clinical characteristics of study population

A total of 139 subjects were enrolled in this study. Plasma samples were collected from all patients in the derivation cohort for miRNA profiling (*n* = 26) and from all patients in the PCR validation cohort (*n* = 113). Among these 139 subjects, 50 patients with clinical suspicion of CAD, defined as symptoms of chest pain or distress due to non-coronary atherosclerosis causes (*e.g.*, gastroesophageal reflux, gastritis, peptic ulcer disease, psychological disturbance, etc.) and angiographic exclusion of coronary atherosclerosis, were enrolled as a control group. Fifty-eight patients with typical UA and angiographically documented CAD were enrolled in the UA group, which included 10 patients with IVUS-confirmed plaque rupture. Thirty-one patients with SA were enrolled in the SA group. All patients enrolled in this study had normal plasma levels of CK-MB (<5 ng/mL) and TNI (<0.04 ng/mL). The clinical characteristics of the study populations are summarized in Table 1 and 2.

**Table 1:** Clinical Characteristics of the Study Population for Circulating MiRNAs Profiling.

	Control cases (n = 13)	UA patients (n = 13)	*P* value
**Baseline data**			
Sex, M/F	5/8	7/6	0.431
Age (yrs)	56±9	61±8	0.159
SBP (mm Hg)	125±12	133±13	0.139
DBP (mm Hg)	76±6	80±11	0.281
BMI (kg/m^2^)	26±5	25±4	0.762
LVEF (%)	69±6	68±6	0.624
HR (bpm)	70±9	70±10	0.935
hs-CRP (mg/dl)	1.18	1.25	0.951
PLT (×10^12^/L)	225±72	209±43	0. 510
Glucose (mmol/l)	5.02±0.83	4.95±0.51	0.806
**Lipid profile**			
LDL cholesterol (mmol/l)	2.31±0.86	2.42±0.56	0.718
HDL cholesterol (mmol/l)	1.02±0.23	0.97±0.30	0.630
TC (mmol/l)	3.96±1.02	3.96±0.60	0.996
**Risk factors**			
Cigarette smoking, %	7.7	23.1	0.593
Hypertension, %	53.8	84.6	0.202
Diabetes mellitus, %	15.4	30.8	0.645
Hyperlipemia, %	53.8	61.5	0.691
**Drug Administration**			
Statin, %	53.8	61.5	0.691
CCB, %	30.8	38.5	>0.99
Beta-blocker, %	38.5	69.2	0.116
Aspirin, %	38.5	61.5	0.239
Clopidogrel, %	30.8	46.2	0.420
ACEI, %	15.4	23.1	>0.99
ARB, %	15.4	15.4	>0.99

BMI, body mass index; SBP, systolic blood pressure; DBP, diastolic blood pressure; LVEF, left ventricular ejection fraction; HR, heart rate; PLT, platelet; CRP, C-reactive protein; LDL, low-density lipoprotein; HDL, high-density lipoprotein; TC, total cholesterol; CCB, calcium channel blocker; ACEI, angiotensin-converting enzyme inhibitor; and ARB, angiotensin receptor blocker. All *P* values represent comparisons between UA patients and controls. Comparisons between groups were performed with Student's *t* test or Mann–Whitney *U* test for continuous variables and with the Fischer exact test or *χ^2^* test for categorical variables.

**Table 2:** Clinical Characteristics of the Study Population for Real-time PCR Validation Cohort.

	Control cases (n = 37)	SA patients (n = 31)	UA patients (n = 45)
**Baseline data**			
Sex, M/F	22/15	18/13	25/20
Age (yrs)	59±6	62±11	63±12
SBP (mm Hg)	127±15	134±22	133±15
DBP (mm Hg)	77±8	80±13	79±10
BMI (kg/m^2^)	26.0±3.4	25.6±2.1	26.5±3.4
LVEF (%)	66±11	69±7	66±7
HR (bpm)	73±11	68±11	72±9
hs-CRP (mg/dl)	1.42	1.80	1.96
PLT (×10^12^/L)	210±58	192±47	213±50
Glucose (mmol/l)	4.95±0.70	5.82±2.42	5.32±0.95
**Lipid profile**			
LDL cholesterol (mmol/l)	2.46±0.78	2.60±0.76	2.66±1.00
HDL cholesterol (mmol/l)	1.16±0.75	1.10±0.50	0.96±0.25
TC (mmol/l)	4.11±0.87	4.34±0.92	4.18±1.08
**Risk factors**			
Cigarette smoking, %	45.9	38.7	46.7
Hypertension, %	56.8	74.2	75.6
Diabetes mellitus, %	16.2	29.0	26.7
Hyperlipemia, %	45.9	45.2	66.7
**Drug Administration**			
Statin, %	48.6	51.6	42.2
CCB, %	32.4	32.3	35.6
Beta-blocker, %	35.1	35.5	51.1
Aspirin, %	35.1	51.6	53.3
Clopidogrel, %	27.0	45.2	28.9
ACEI, %	16.2	19.4	24.4
ARB, %	16.2	25.8	11.1

BMI, body mass index; SBP, systolic blood pressure; DBP, diastolic blood pressure; LVEF, left ventricular ejection fraction; HR, heart rate; PLT, platelet; CRP, C-reactive protein; LDL, low-density lipoprotein; HDL, high-density lipoprotein; TC, total cholesterol; CCB, calcium channel blocker; ACEI, angiotensin-converting enzyme inhibitor; and ARB, angiotensin receptor blocker.

### Expression profile of miRNAs in the plasma of UA patients

**Figure 2:**
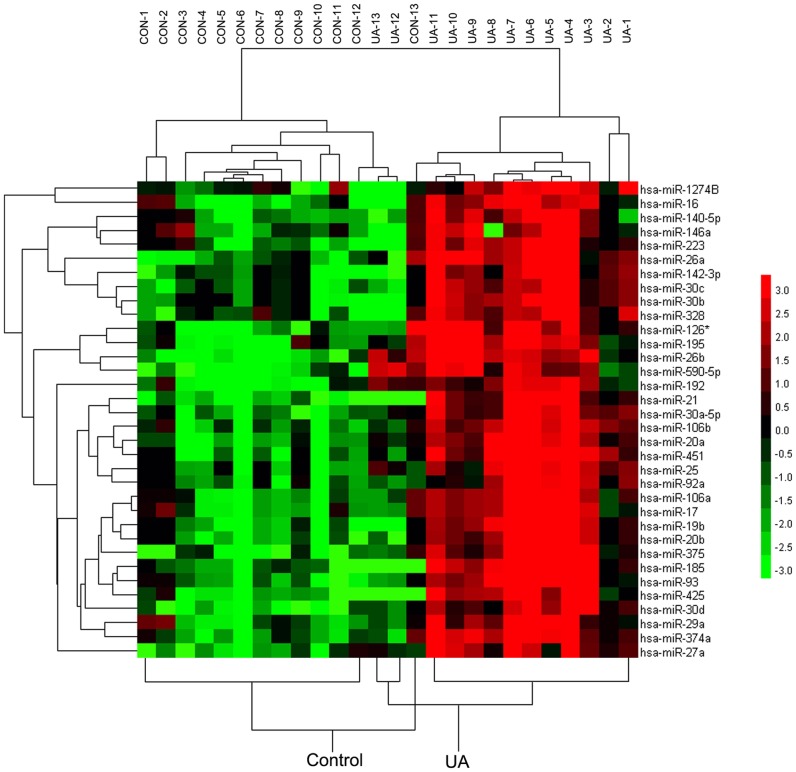
Profile of circulating miRNAs in UA patients (*n* = 13) and controls (*n* = 13). Heat map illustrates the levels of significantly changed miRNAs (fold change >8 and FDR <0.0001%) in UA patients compared with controls. Color intensity is scaled within each row, such that the highest expression value corresponds to bright red and the lowest to bright green.

We analyzed the miRNA expression profiles in the plasma of patients with chest pain or distress attributable to non-cardiac causes (control group, *n* = 13) and patients with typical UA (UA group, *n* = 13) using TLDA (Table 3 and Figure 2). Unsupervised hierarchical clustering based on miRNA expression clearly separated UA patients from control cases. Analysis of array data with SAM identified 34 significantly deregulated miRNAs (fold change >8 and FDR <0.0001%). All of these miRNAs were upregulated in UA patients compared to controls.

**Table 3:** Deregulated miRNAs in the circulation of UA patients compared with controls.

MiRNA ID	Score	Fold Change	q-value(%)
***miR-106b/25 cluster***			
hsa-miR-106b	2.506	15.17	0.000
hsa-miR-25	2.427	19.28	0.000
hsa-miR-93	2.034	20.32	0.000
***miR-17/92a cluster***			
hsa-miR-17	2.939	8.830	0.000
hsa-miR-19b	3.322	16.30	0.000
hsa-miR-20a	3.132	18.86	0.000
hsa-miR-92a	2.576	12.88	0.000
***miR-21/590-5p family***			
hsa-miR-21	3.092	23.71	0.000
hsa-miR-590-5p	1.892	14.48	0.000
***miR-106a/20b cluster***			
hsa-miR-106a	3.055	10.62	0.000
hsa-miR-20b	2.875	12.78	0.000
***miR-16/195 family***			
hsa-miR-16	2.997	9.023	0.000
hsa-miR-195	2.660	10.26	0.000
***miR-26a/b family***			
hsa-miR-26a	2.323	15.25	0.000
hsa-miR-26b	3.383	14.22	0.000
***miR-30 family***			
hsa-miR-30a-5p	2.729	16.47	0.000
hsa-miR-30b	2.063	15.97	0.000
hsa-miR-30c	1.955	16.33	0.000
hsa-miR-30d	1.975	12.23	0.000
***Other miRNAs***			
hsa-miR-126*	2.304	16.01	0.000
hsa-miR-1274b	1.791	13.75	0.000
hsa-miR-140-5p	1.814	12.26	0.000
hsa-miR-142-3p	2.248	12.86	0.000
hsa-miR-146a	2.326	8.845	0.000
hsa-miR-185	1.983	17.54	0.000
hsa-miR-192	3.279	8.702	0.000
hsa-miR-223	2.556	9.644	0.000
hsa-miR-27a	1.874	13.25	0.000
hsa-miR-29a	2.062	9.025	0.000
hsa-miR-328	1.867	10.37	0.000
hsa-miR-374a	2.461	13.43	0.000
hsa-miR-375	2.177	9.990	0.000
hsa-miR-425	2.740	18.87	0.000
hsa-miR-451	2.957	24.20	0.000

Data include miRNAs showing significant change (>8-fold change and FDR <0.0001%) in plasma of UA patients (*n* = 13) compared to controls (*n* = 13).

Among these differentially expressed miRNAs, several were derived from close genomic loci (< 10 kb), which were defined as miRNA clusters, such as the miR-106b/25/93 cluster and miR-17/19b/20a/92a cluster. Several deregulated miRNAs were classified into the same miRNA family, such as the miR-21/590-5p family. Members within the same miRNA family share 5′ seed sequences, which are highly conserved 7- or 8-mer sequences within miRNAs that establish target specificity. The miRNA family members tend to be coexpressed and may exert similar biological functions. These findings indicate that the dysregulation of miRNA expression in the circulation of UA patients occurred in a regulated manner. We selected 7 miRNAs (*i.e.*, miR-106b, miR-25, miR-92a, miR-21, miR-590-5p, miR-126*, and miR-451) for further validation by quantitative RT-PCR in a larger independent patient cohort. These miRNAs were selected based on their expression difference between UA patients and controls (fold change >8 and FDR <0.0001%), abundance in the circulation (expressed in at least 21/26 samples), previously reported biological functions relevant to vulnerable plaque pathogenesis, and representation of different miRNA families and clusters.

### Quantitative RT-PCR validation of profiling data

**Figure 3:**
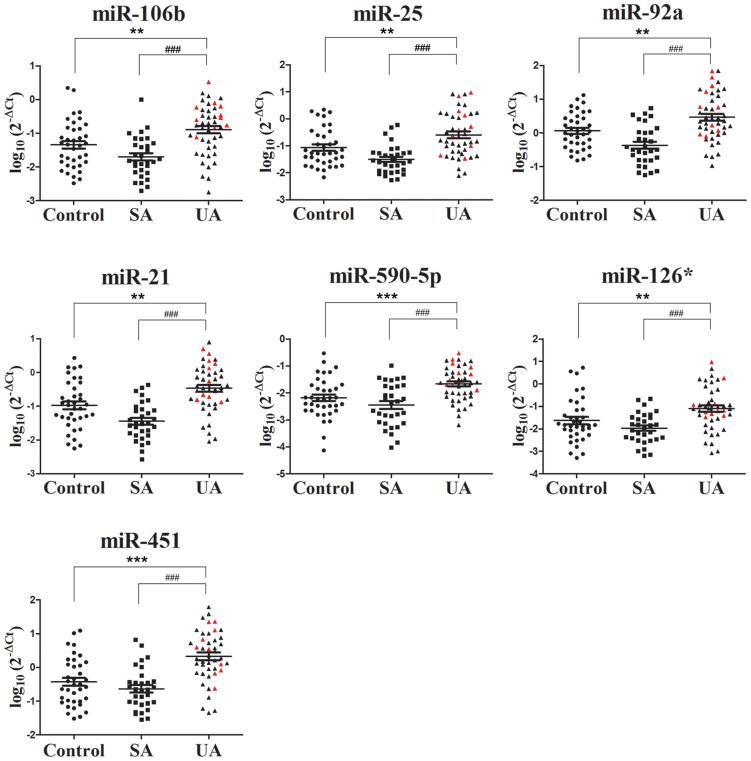
Circulating miRNAs expression in the validation cohort. Expression of selected miRNAs were analyzed in controls (*n* = 37), SA patients (*n* = 31), and UA patients (*n* = 45) by quantitative PCR. Red points in UA group indicate UA patients with IVUS-confirmed plaque rupture. Data represent the mean ± SEM. ** *P*<0.01, *** *P*<0.001 compared to control group; ## *P*<0.01, ### *P*<0.001 compared to SA group.

**Figure 4:**
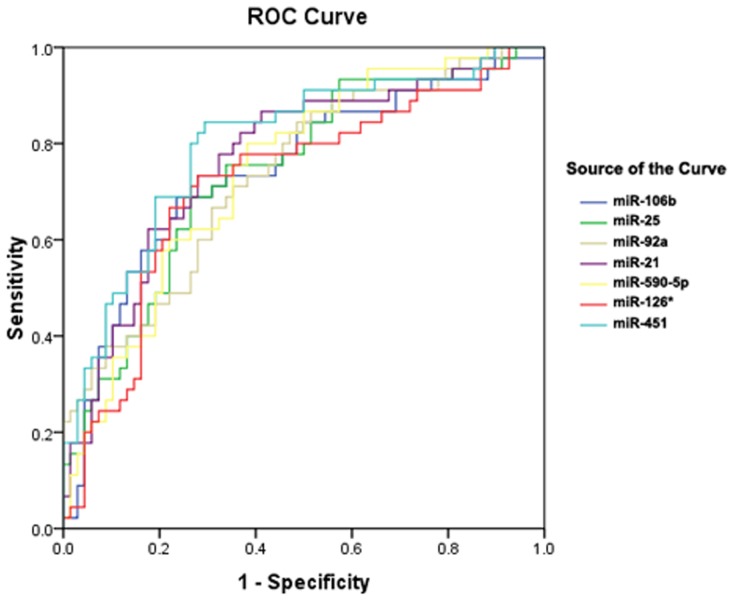
Receiver–operator characteristics (ROC) curves for selected miRNAs in validation cohort. ROC curves regarding diagnostic power to distinguish UA patients from non-UA cases for 7 selected miRNAs in the validation cohort were shown.

The expression of 7 selected miRNAs was validated in an independent cohort (45 UA patients, 31 SA patients, and 37 controls) by real-time RT-PCR. Consistent with the profiling data, the levels of these 7 miRNAs were increased (*P*<0.01) in UA patients compared to either controls or SA patients (Figure 3). This finding indicated that the circulating miRNA levels could distinguish vulnerable CAD patients from patients with more benign forms or non-cardiac chest pain. The area under the receiver–operator characteristic curve (AUC) was determined for selected miRNA to distinguish UA cases from non-UA cases in the validation cohort (Figure 4 and Table 4). The cut-off values and their corresponding sensitivity and specificity are shown in Table 4.

**Table 4:** The area under receiver–operator characteristic curve (AUC) and cut-off point for the levels of selected miRNA in PCR validation cohort.

MiRNAID	AUC	95%CI	*P*	Cut-offpoint	Sensitivity	Specificity
miR-106b	0.743	(0.647,0.839)	<0.001	−1.131	68.9%	76.5%
miR-25	0.741	(0.648,0.834)	<0.001	−1.029	68.9%	73.5%
miR-92a	0.735	(0.642,0.828)	<0.001	−0.170	84.4%	51.5%
miR-21	0.770	(0.680,0.859)	<0.001	−1.076	86.7%	58.8%
miR-590-5p	0.742	(0.651,0.833)	<0.001	−2.153	80.0%	61.8%
miR-126*	0.720	(0.620,0.819)	<0.001	−1.474	73.3%	72.1%
miR-451	0.799	(0.714,0.885)	<0.001	−0.276	84.4%	70.6%

The AUC was determined for selected miRNA to distinguish UA from non-UA cases in the validation cohort. The cut-off values and their corresponding sensitivity and specificity are shown. The value of cut-off point for each circulating miRNA is expressed as log10 (2^−ΔCt^). 95% CI, 95% confidence interval.

To establish independent associations, we performed logistic regression analysis with UA as the dependent variable and including established risk factors (*e.g.*, age, sex, hypertension, dyslipidemia, diabetes mellitus, and smoking status), the use of statins and anti-platelet drugs, and miRNA levels. After adjustment for risk factors and the use of statins and anti-platelet drugs, the circulating levels of miR-106b, miR-25, miR-92a, miR-21, miR-590-5p, miR-126*, and miR-451 remained independently associated with UA (all *P*<0.05; Table 5).

**Table 5:** Logistic regression analysis of circulating miRNA levels in PCR validation cohort.

	UA versus SA			UA versus Control		
MiRNAID	OR	95% CI	*P*	OR	95% CI	*P*
miR-106b	7.024	(2.728,18.082)	<0.001	2.389	(1.158, 4.927)	0.018
miR-25	7.367	(2.713,20.004)	<0.001	2.036	(1.048, 3.955)	0.036
miR-92a	10.170	(3.437,30.094)	<0.001	2.611	(1.110, 6.144)	0.028
miR-21	8.828	(3.302,23.603)	<0.001	2.488	(1.173, 5.277)	0.017
miR-590-5p	6.314	(2.534,15.729)	<0.001	2.678	(1.226, 5.849)	0.013
miR-126*	3.457	(1.730,6.910)	<0.001	1.882	(1.140, 3.108)	0.013
miR-451	6.664	(2.774,16.010)	<0.001	4.609	(2.171, 9.782)	<0.001

Logistic regression analysis comparing circulating miRNA levels between controls (*n* = 37), SA patients (*n* = 31), and UA patients (*n* = 45). Levels of the 7 selected circulating miRNAs were independently associated with UA after adjustment for age, sex, hypertension, dyslipidemia, diabetes mellitus, smoking status, and the use of statins and anti-platelet drugs. OR, odds ratio; 95% CI, 95% confidence interval.

### Disease-specific miRNA expression pattern in the circulation of UA patients

**Figure 5:**
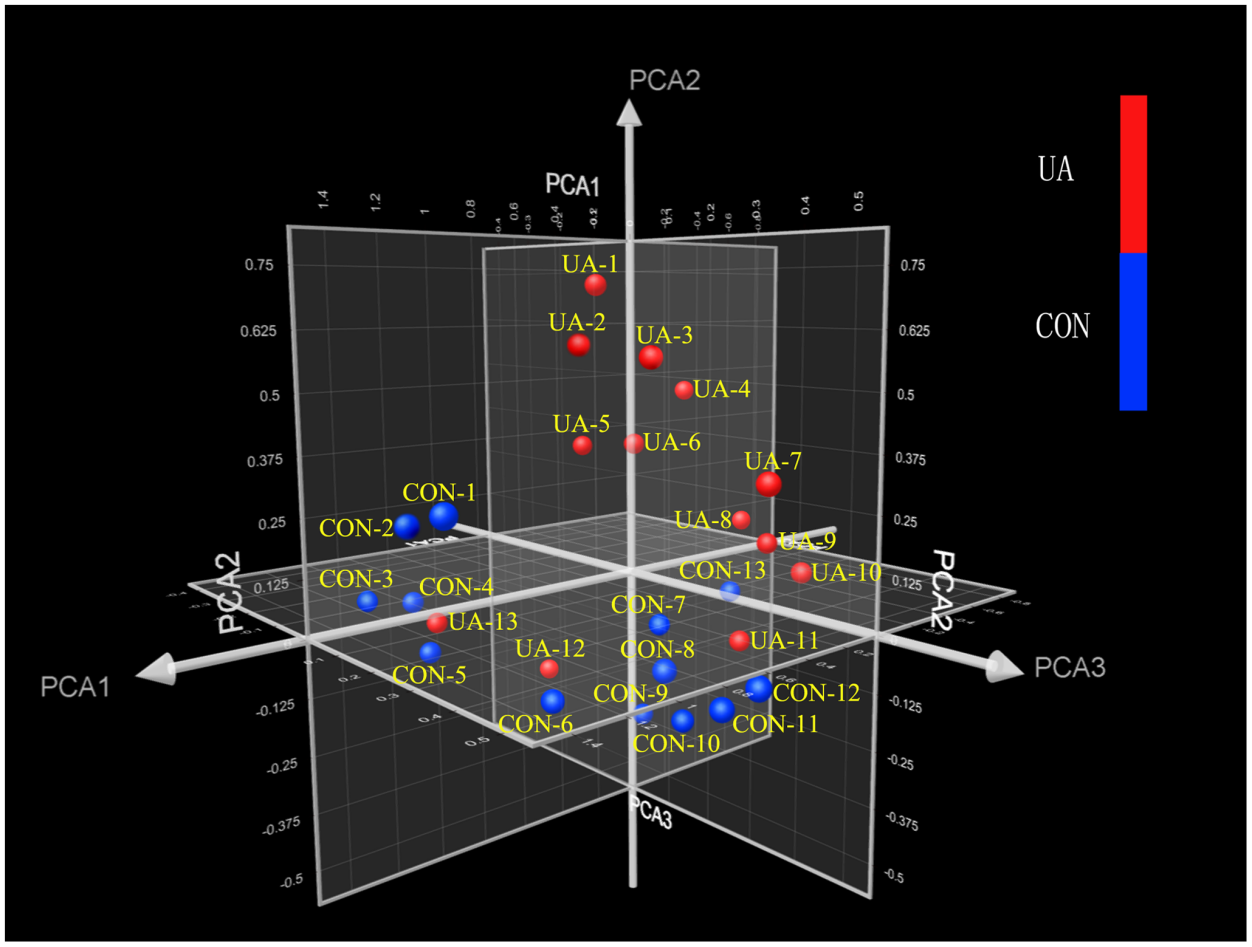
Principle component analysis (PCA) of circulating miRNA profiling in the derivation cohort. The miRNA expression profile was reduced to three main principal components. PCA showed that most UA cases (84.6%, 11/13) could be correctly classified from the control group (CON).

**Figure 6:**
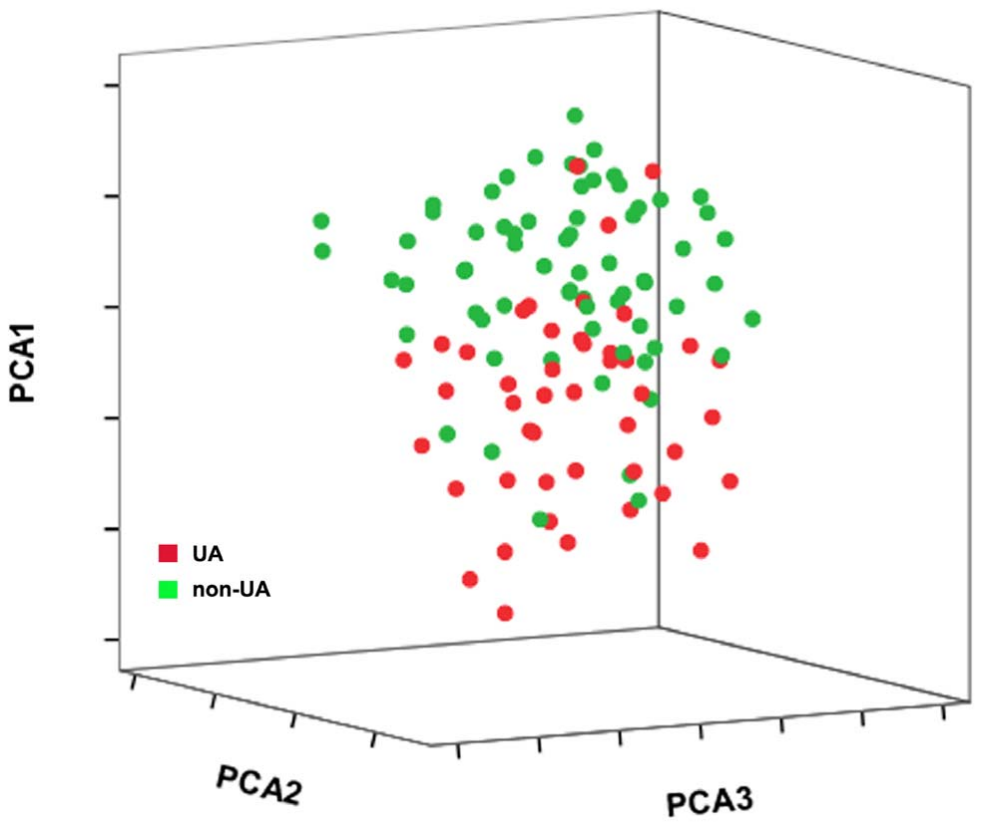
Principle component analysis (PCA) of circulating miRNA expression in the validation cohort. PCA decomposition of the 7 selected miRNAs (miR-106b, miR-25, miR-92a, miR-21, miR-590-5p, miR-126*, and miR-451) could distinguish most UA cases (84.4%, 38/45) from the non-UA cases in the PCR validation cohort.

Principal component analysis (PCA) is a technique for extracting the multivariate data features by reducing the number of dimensions. To determine whether the circulating miRNA profile can differentiate individuals with unstable CAD from patients with non-cardiac chest pain, we used PCA to reduce the overall miRNA expression data to three uncorrelated principal components. The principal components are ordered according to the amount of variance they explain. In 3-dimension PCA graph, the miRNA expression data are represented as a cloud of points in three dimensional space. PCA showed that 84.6% (11/13) of UA patients could be correctly classified from control cases (Figure 5). In addition, we conducted PCA analysis in the PCR validation cohort and found that PCA decomposition of the 7 selected miRNAs could distinguish most UA cases (84.4%, 38/45) from the non-UA cases in the PCR validation cohort (Figure 6). These findings indicated that the circulating miRNA signature could be used for the identification of unstable CAD patients.

**Figure 7:**
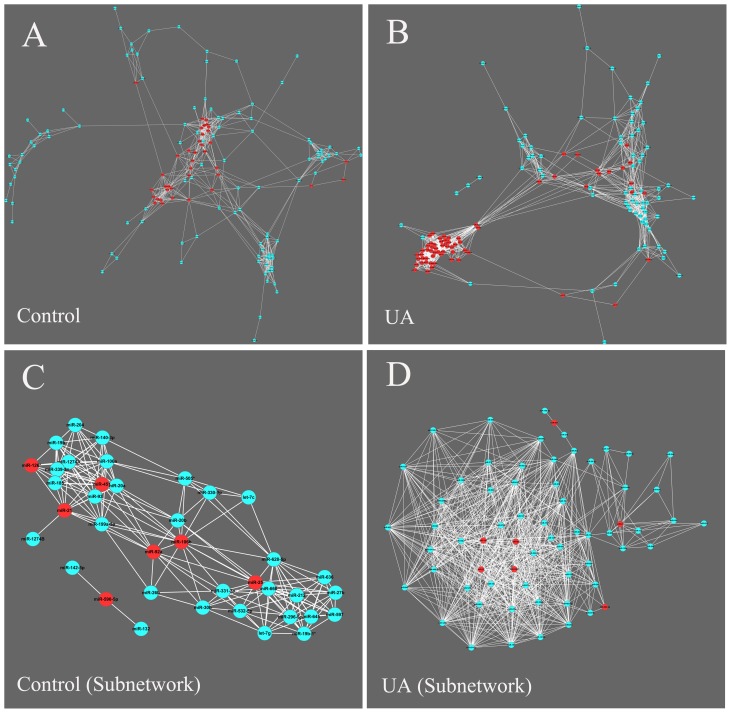
Coexpression network analysis of circulating miRNAs in UA patients and controls. (A–B) Coexpression network constructed with all detected miRNAs. Nodes represent individual miRNAs, and edges represent coexpression relationships between miRNA pairs. PCR-validated miRNAs (miR-106b, miR-25, miR-92a, miR-21, miR-590-5p, miR-126*, and miR-451) and their first neighbors are labeled with red nodes. (C–D) Subnetwork constructed with PCR-validated miRNAs (red nodes) and their first neighbors.

We performed a weighted and undirected miRNA coexpression network analysis to investigate the interactions among miRNAs. The miRNA coexpression networks were built with the Cytoscape v.2.8.2 software package, according to the normalized miRNA expression levels. For each miRNA pair, we calculated the Pearson correlation coefficient. Only high and statistically significant correlation pairs (Pearson correlation coefficient ≥0.79, *P*<0.02) were chosen to construct the network, in which nodes represented individual miRNAs and edges represented coexpression relationships between miRNA pairs. The coexpression pattern of the UA group (140 nodes, 1167 edges) differed from that of the non-cardiac control group (141 nodes, 618 edges) (Figure 7A and 7B).

We used the PCR-validated miRNAs (*i.e.*, miR-106b, miR-25, miR-92a, miR-21, miR-590-5p, miR-126*, and miR-451) and their first neighbors to create a subnetwork (Figure 7C and 7D). The network of the UA group (65 nodes, 660 edges) exhibited a specific spatial coexpression pattern that completely differed from that of the control group (38 nodes, 183 edges). Only 18 links were shared between the two groups in the subnetwork, indicating a disease-mediated change in circulating miRNA expression.

### Comparison of miRNA expressions in MPs from plasma samples

**Figure 8:**
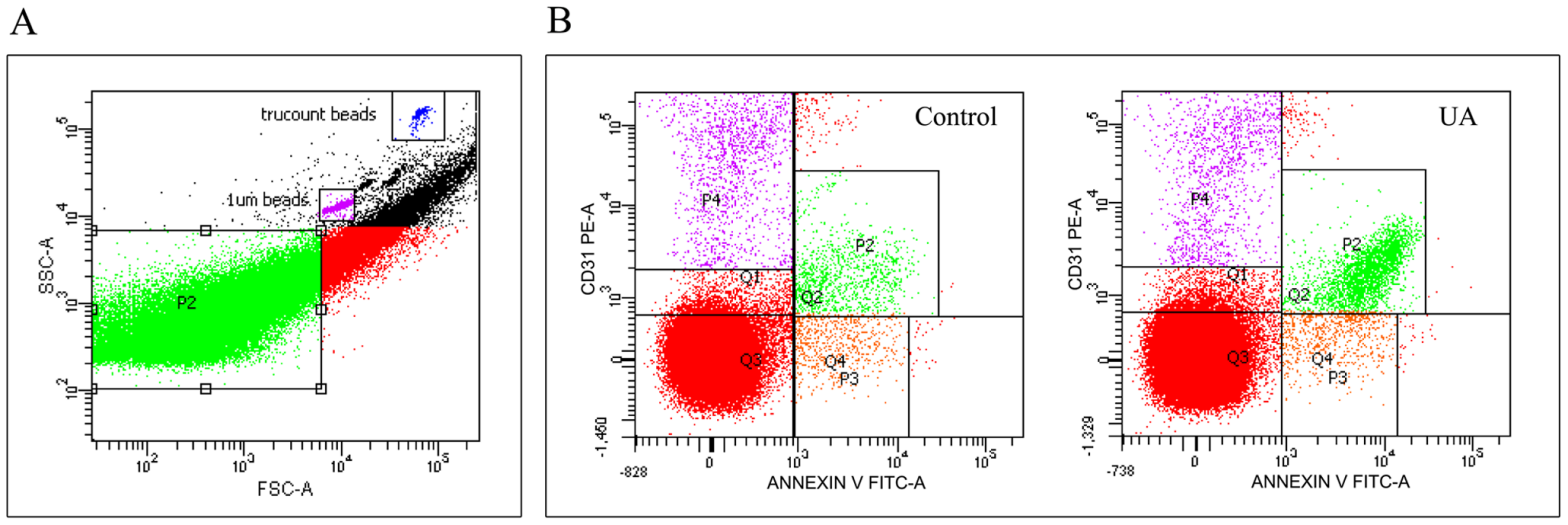
Characterization of microparticles (MPs) from plasma of control and UA patients by flow cytometry. (A) Trucount beads and calibrator beads (1 µm in diameter) were gated in the upper two windows respectively. MPs are particles with size between 0.1 and 1 µm and gated in window P2. (B) Representative flow cytometry plot displaying MPs from the plasma of controls and UA patients stained with AnnexinV-FITC and anti-CD31-phycoerythrin. Annexin V^+^ CD31^+^ MPs were gated in window P2.

**Figure 9:**
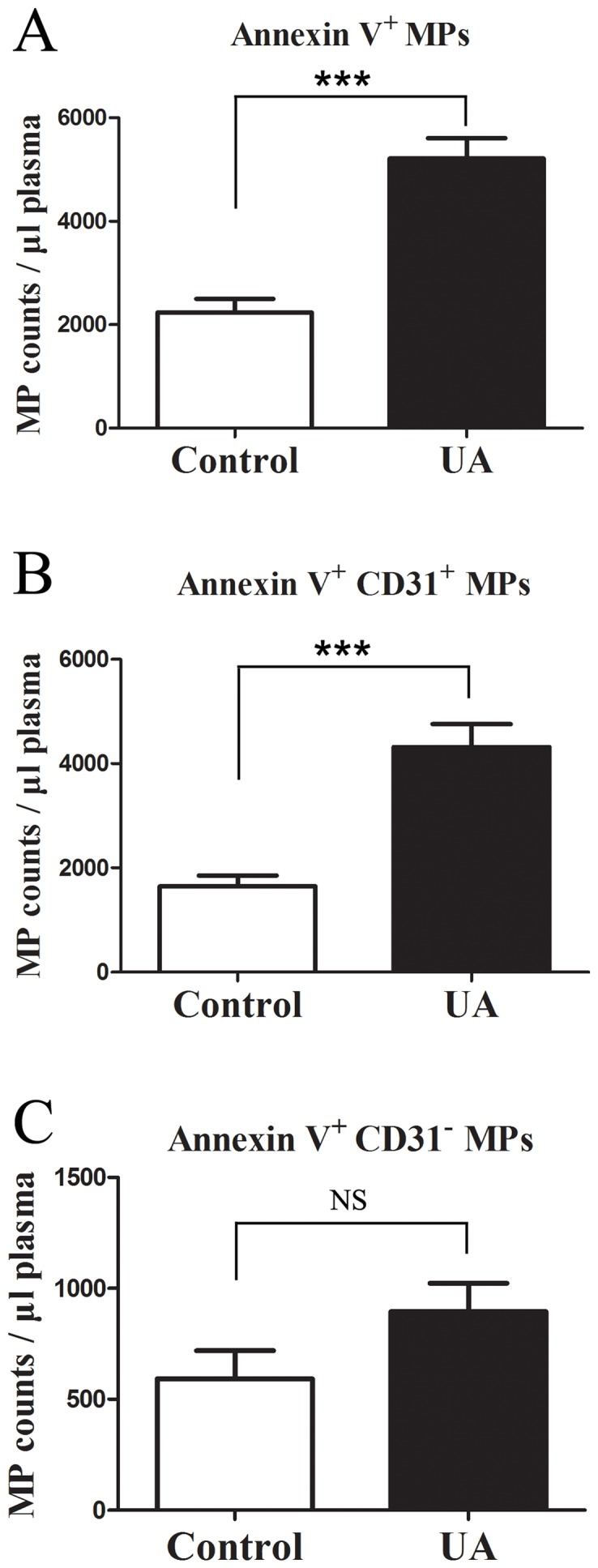
Comparison of microparticles (MPs) counts isolated from the plasma of UA patients (*n* = 5) and controls (*n* = 5). (A) Comparison of Annexin V^+^ MP counts in the plasma of UA patients and controls. (B) Comparison of Annexin V^+^ CD31^+^ MP counts in the plasma of UA patients and controls. (C) Comparison of Annexin V^+^ CD31^−^ MP counts in the plasma of UA patients and controls. MP counts were determined by flow cytometry. Data represent the mean ± SEM. *** *P*<0.001; NS, non-significant.

To explore whether these upregulated miRNAs in the circulation of UA patients are derived from MPs, we analyzed the miRNA expression in MPs isolated from the plasma samples of UA patients (*n* = 5) and controls (*n* = 5). Isolated MPs were characterized by flow cytometry as the particles of 0.1 to 1 μm in size and specifically detected with Annexin V labeling (Figure 8A). We found that Annexin V^+^ MPs (5213 ± 882/µL plasma) were increased in the plasma samples of UA patients compared to controls (2240 ± 585/µL plasma; *P*<0.001) (Figure 9A).

Next, we decided to determine the potential cellular origin(s) of these Annexin V^+^ MPs. Using immunolabeling, we showed that the majority of the increased MPs in plasma of UA patients was Annexin V^+^ CD31^+^, which have been previously reported to be released from endothelial cells (ECs) and platelets [14]–[16]. Annexin V^+^ CD31^+^ MPs (4318±988/µL plasma) were significantly upregulated in UA patients compared with controls (1646±462/µL plasma; *P*<0.001) (Figure 8B, Figure 9B), while Annexin V^+^ CD31^−^ MPs counts in UA patients (895±287/µL plasma) were not significantly higher than controls (594±280/µL plasma; *P*>0.05) (Figure 9C).

**Figure 10:**
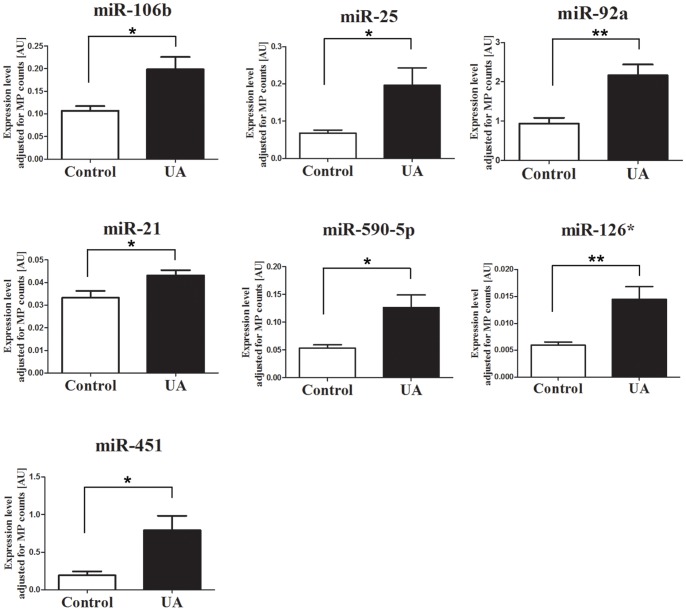
Comparison of miRNA expression in microparticles (MPs) isolated from the plasma of UA patients (*n* = 5) and controls (*n* = 5). Expression levels of selected miRNAs were analyzed by quantitative PCR. Data represent the mean ± SEM. * *P*<0.05; ** *P*<0.01.

Next, we detected the miRNA expression levels in MPs isolated from the plasma of UA patients and controls. The 7 selected miRNAs were all significantly (*P*<0.05) increased in UA patients compared with the controls after being adjusted to MPs counts (Figure 10). Taken together, these data indicate that the upregulation of selected miRNAs in the circulation of UA patients may be at least partially due to the increased level of MPs, especially Annexin V^+^ CD31^+^ MPs.

## Discussion

In this study, we identified a distinct plasma miRNA expression pattern in vulnerable CAD. Specifically, we found a circulating miRNA signature, including the miR-106b/25 cluster, miR-17/92a cluster, miR-21/590-5p family, miR-126*, and miR-451, upregulated in vulnerable CAD patients compared to patients with stable angina or with non-cardiac chest pain, which are conditions that need to be discriminated in clinical practice. To emulate the real-world practice, we chose patients with non-cardiac chest pain rather than healthy volunteers as the control group. Among these upregulated miRNAs, none were the known cardiac muscle-enriched miRNAs that are released into the circulation because cardiac injury occurs after acute MI [11]. Several vascular and inflammation-associated miRNAs that were previously reported to be downregulated in stable CAD patients [12], such as hsa-miR-21, hsa-miR-17, hsa-miR-20a, and hsa-miR-92a, were found to be upregulated in unstable CAD patients in our results. This finding indicates that the miRNA profile in UA patients displays a completely different expression pattern from the pattern in acute MI or stable CAD patients.

PCA of the miRNA profiling data and PCR validation data revealed the potential value of the circulating miRNA expression profile in the diagnosis of vulnerable patients. We used a coexpression network analysis to evaluate the changes of individual miRNAs in the context of the overall miRNA network, which offers additional insights compared to the evaluation of individual miRNAs alone [17]. Different patterns for the coexpression networks were obtained for the UA and the control groups. Because the miRNA coexpression pattern can reflect cooperative relationships among different miRNA pairs, the comprehensive transition of the circulating miRNA coexpression mode indicates the dysregulation of miRNA coordination in vulnerable CAD. For example, the first neighbors of known inflammation-associated miRNAs (*i.e.*, miR-155, miR-144*, miR-27a, miR-122, and miR-125b) [18]–[22] present in the subnetwork of UA group, but not in control group. This finding indicates that the cooperation of these miRNAs may play a role in the pathogenesis of vulnerable CAD.

Circulating miRNAs have been shown to modulate target mRNA expression in recipient cells [9], [23]. To understand the biological roles of these deregulated circulating miRNAs in vulnerable CAD, we predicted their target genes with the TargetScan algorithm (Table S1). We found that these differentially expressed miRNAs (*i.e.*, miR-106b, miR-25, miR-92a, miR-21/590-5p, miR-126* and miR-451) may affect several aspects of vulnerable plaques, such as inflammation, hypoxia, angiogenesis, apoptosis, and extracellular matrix (ECM) degradation (Table S1). They may regulate several key signaling pathways in vulnerable plaque pathogenesis, such as pathways involving transforming growth factor-β (TGF-β), toll-like receptor-4 (TLR-4), hypoxia-inducible factor 1α (HIF-1α), and peroxisome proliferator-activated receptor-α (PPAR-α).

The miR-106b/25 and miR-17/92a clusters are paralogs with similar expression patterns and functional cooperation. These miRNAs have been shown to be involved in the pathogenesis of vulnerable plaque through regulating TGF-β signaling [24]. TGF-β signaling is an important mediator of the balance between inflammation and fibrosis, which are critical processes in the transition from stable to rupture-prone and ruptured atherosclerotic plaques [25]. The miR-17/92a cluster has been shown to control angiogenesis, which plays important roles in plaque destabilization and rupture [26]. Meanwhile, miR-21 can attenuate plaque stability by downregulating the expression of matrix metalloproteinase inhibitors, thereby leading to matrix metalloproteinase activation and subsequent ECM degradation [27]. Circulating miRNAs have been shown to play important roles by targeting the genes of vascular ECs [28], suggesting that the dysregulation of circulating miRNA levels in unstable CAD patients may contribute to vulnerable plaque progression.

Interestingly, we found that the expression of miR-451, which is enriched in the blood platelets of premature CAD patients [29], was increased in the circulation of UA patients. Recent studies demonstrated the role of miR-451 in the modulation of proinflammatory cytokine production (*e.g*., macrophage migration inhibitory factor) and the PI3K/AKT pathway [30], [31]. The question of whether miR-451 plays a similar role in vulnerable CAD requires further investigation.

Notably, miR-126*, which was deregulated in UA patients, is processed from the same miRNA precursor as miR-126. Both of these miRNAs are specifically expressed in ECs [6], and miR-126 has been implicated in many critical process associated with plaque rupture (*e.g*., EC functions, angiogenesis, and inflammation) [7]. However, little is known about the function of miR-126*, which may be due to the less abundance of miR-126* compared with miR-126 in ECs. The level of miR-126*, but not miR-126, was significantly increased in the circulation of UA patients compared to control cases. This finding suggests that the molecular mechanisms regulating the release of miR-126* from ECs may be involved in the pathogenesis of vulnerable CAD. Kuchenbauer *et al*. recently reported that miRNA*s may be more abundant than previously assumed, implying a functional role for highly expressed miRNA*s [32].

MPs were reported to be one of the major carriers of miRNAs in circulation [9], [10]. To explore the potential origins of these upregulated miRNAs in the circulation of UA patients, we isolated MPs from plasma and detect the levels of MPs. Annexin V^+^ MPs were found to be significantly increased in the plasma of UA patients compared to controls. We further detected miRNA levels in MPs and found that MPs shared the similar miRNA signature as plasma, that is the increased expression of miR-106b, miR-25, miR-92a, miR-21/590-5p, miR-126* and miR-451 in UA patients compared with controls. These findings indicate that MPs may contribute to the deregulation of circulating miRNA expression in vulnerable CAD patients. MPs are released from various cell types, such as ECs, leucocytes, erythrocytes, and platelets and they expressed different cell surface markers according to their cell origins [33]. In this study, we showed that the majority of increased MPs in the plasma of UA patients were Annexin V^+^ CD31^+^ MPs, which are previously reported to origin from ECs and platelets [14]–[16]. This indicated that the upregulated miRNAs in the circulation of vulnerable CAD patients may be derived from the systemically activated/apoptotic ECs and stimulated platelets.

UA is not a specific disease, but rather constitutes a clinical syndrome subset of acute coronary syndrome, which is caused by different etiological mechanisms (*e.g.*, atherosclerotic plaque, coronary focal spasm, coronary artery dissection, microvascular disease, syndrome X, steal, etc). The most common cause of UA is reduced myocardial perfusion resulting from coronary artery narrowing due to a vulnerable atherosclerotic plaque. Therefore, in the current study, we focused on patients with this type of UA and enrolled patients with typical UA and angiographically documented CAD. The signature of circulating miRNAs in other types of UA need to be addressed in future trials.

In conclusion, we have identified a distinct miRNA expression signature in the plasma of vulnerable CAD patients, which discriminates these high-risk patients from individuals with SA or non-cardiac chest pain. In addition, Annexin V^+^ CD31^+^ MPs may contribute to the elevated expression of these miRNAs in the circulation of vulnerable CAD patients. Thus, this circulating miRNA signature may be used as a novel biomarker for the diagnosis of vulnerable CAD, although its sensitivity and specificity need to be further examined in a larger clinical cohort.

## Supporting Information

Table S1 **Potential and validated gene targets of differentially expressed circulating miRNAs in UA patients and controls.**
(DOC)

Protocol S1 **The detailed study design for this observational trial was shown in Protocol S1.**
(PDF)

Checklist S1 **Supporting CONSORT checklist, including items when reporting a observational trial, were summarized in Checklist S1.**
(PDF)
