# Supplementary material for: Signature of Circulating MicroRNAs as Potential Biomarkers in Vulnerable Coronary Artery Disease
Source: PLoS One. 2013 Dec 5;8(12):e80738. doi: 10.1371/journal.pone.0080738 (PMC3855151; doi:10.1371/journal.pone.0080738)
Supplement: Table S1 — Potential and validated gene targets of differentially expressed circulating miRNAs in UA patients and controls. (DOC) [file pone.0080738.s001.doc]

**Supplementary Table S1. Potential and validated gene targets of differentially expressed circulating miRNAs in UA patients and controls**.

| **miRNA**  **name** | **Predicted Targets** | **Validated Targets** | **Potential function of target gene in the pathogenesis of vulnerable plaque** |
| --- | --- | --- | --- |
| **miR-106b** | MMP2 (matrix metalloproteinase 2)^1^ |  | Degradating of extracellular matrix (type IV collagen )and contributing to plaque rupture |
|  |  | CDKN1A/ p21 (cyclin-dependent kinase inhibitor 1A )^2^ | Key regulatory protein of cell senescent and inhibitor of cell cycle |
|  |  | TGF-β (transforming growth factorβ) signaling^3^ | Balancing of two key modulators of plaque rupture: inflammation and fibrosis |
|  |  | BCL2L11 (BCL2-like 11)^3^ | Cell apoptosis facilitator |
|  |  | ABCA1 (ATP-binding cassette transporter A1)^4^ | Promoting cholesterol efflux |
|  |  | TβR II (TGF-β type II receptor)^5^ | Regulating TGF-β signaling pathway |
|  | LDLR (low density lipoprotein receptor)^1^ |  | Regulation of lipid metabolism and formation of foam cells within plaque |
|  | CXCL14 (chemokine C-X-C motif ligand 14)^1^ |  | Chemotaxis of inflammatory cells such as activated macrophages and T cells |
|  | KLF11 (Kruppel-like factor 11)^1^ |  | Regulating TGF-β signaling |
|  | HIF1A (hypoxia inducible factor 1, alpha subunit)^1^ |  | Inducing angiogenesis |
|  |  | VEGFA (vascular endothelial growth factor A)^6^ | Inducing angiogenesis |
|  |  | E2F1 (E2F transcription factor 1)^7^ | Controling endothelial cell proliferation |
|  |  | MAPK14 (mitogen-activated protein kinase 14)^8^ | A pro-inflammatory mediator |
|  |  | STAT3 (signal transducer and activator of transcription 3)^8^ | Key signaling pathway involved in inflammation and oxidative stress |
| **miR-25** | NOX4 (NADPH oxidase 4)^1^ |  | Regulating ROS production |
|  | KLF2 (Kruppel-like factor 2)^1^ |  | Regulating endothelial cell function and activation, modulating macrophage and neutrophil adhesion, monocyte differentiation |
|  |  | BCL2L11 (BCL2-like 11)^2^ | Induction of cell apoptosis |
|  |  | Wnt signaling^9^ | Promoting cell proliferation |
|  | ITGA5 (integrin, alpha 5)^1^ |  | Promoting inflammation cell adhesion |
|  |  | BIM (BCL-2-interacting mediator of cell death)^10^ | Cell apoptosis facilitator |
|  | RGS (regulator of G-protein signaling) 3^1^ |  | Blocking the signal transduction of chemokine receptors, inhibiting activation of ERK 1/2 on endothelial |
|  | COLIA2 (alpha 2 gene of type I collagen)^1^ |  | A component of extracellular matrix |
|  |  | DR4 (TRAIL Death Receptor-4)^11^ | Promoting cell apoptosis |
| **miR-92a** |  | KLF 2,4 (Kruppel-like factor 2,4)^12^ | Regulating endothelial cell function, protecting endothelium against atherogenesis |
|  | COL5A1 (collagen, type V, alpha 1)^1^ |  | A component of extracellular matrix |
|  | TRAF3 (TNF receptor- associated factor 3)^1^ |  | Inducing cell inflammation |
|  |  | TGF-β (transforming growth factor β) signaling^13^ | Regulating cell proliferation and collagen synthesis |
|  | AGGF1 (angiogenic factor with G patch and FHA domains 1)^1^ |  | Inducing angiogenesis |
|  |  | BIM (BCL-2-interacting mediator of cell death)^14^ | Cell apoptosis facilitator |
|  | PPARGC1B (peroxisome proliferator-activated receptor gamma, coactivator 1 beta)^1^ |  | Regulating intracellular lipid metabolism, inflammatory signaling pathways |
|  | ITGA5 (integrin, alpha 5)^1^ |  | Promoting inflammation cell adhesion |
|  |  | HIF1A (hypoxia inducible factor 1, alpha subunit)^15^ | Inducing angiogenesis |
|  | HIPK3 (homeodomain interacting protein kinase 3)^16^ |  | Regulating cell apoptosis |
|  | MYLIP (myosin regulatory light chain interacting protein)^16^ |  | Regulator of the LDL receptor (LDLR) pathway |
| **miR-21/**  **590-5p** |  | TIMP 3 (tissue inhibitor of metalloproteinase 3)^17^ | Matrix metalloproteinase inhibitors |
|  |  | PDCD4 (programmed cell death 4)^18^ | A proinflammatory protein modulating TLR4/NF-κB pathway |
|  | TGFBI (transforming growth factor-β-induced)^1^ |  | Modulating cell adhesion and fibrosis |
|  | TβR II (TGF-β type II receptor)^1^ |  | Regulating TGF-β signaling pathway |
|  | CCL (chemokine ligand) ^1^ |  | Chemotaxis of inflammatory cells, such as monocytes and T cells |
|  | KLF 6 (Kruppel-like factor 6)^1^ |  | Activating IGF1R (insulin growth factor 1 receptor) pathway |
|  |  | PPARA (peroxisome proliferator-activated receptor alpha)^19^ | Regulating intracellular lipid metabolism, fibrosis, inflammatory signaling pathways |
|  | COL4A1 (collagen, type IV, alpha 1)^1^ |  | A component of extracellular matrix |
|  | IL12A (interleukin 12A)^1^ |  | Inducing inflammatory response |
|  | LOX-1(lectin-type oxidized LDL receptor 1)^1^ |  | A scanvenger receptor for oxidized low-density lipoprotein (ox-LDL) , involved in oxidative stress and inflammatory response |
|  |  | Bcl-2 (B-cell lymphoma 2)^20^ | Regulating cell apoptosis |
|  |  | PTEN (phosphatase and tensin homolog deleted on chromosome ten)^20^ | Controling cell proliferation |
|  |  | HIF1A (hypoxia inducible factor 1, alpha subunit)^21^ | Inducing angiogenesis |
|  |  | RhoB (ras homolog gene family, member B)^22^ | Regulating endothelium function, angiogenesis and inflammation |
|  |  | STAT3 (signal transducer and activator of transcription 3)^23^ | Key signaling pathway involved in inflammation and oxidative stress |
| **miR-126*** |  | ABCA5 (ATP-binding cassette transporter A5)^24^ | Regulating cellular cholesterol homeostasis and atherosclerotic lesion development |
|  |  | PDGF-D (Platelet-derived growth factor-D)^24^ | Regulating MMP activity and influencing monocyte migration |
|  |  | IL-7 (Interleukin-7)^24^ | A regulator of T-cell homeostasis and inflammation in atherogenesis and the promotion of clinical instability in coronary artery disease |
|  |  | IL-17 (Interleukin-17)^24^ | Involved in many inflammatory processes and plaque stability |
|  |  | ADAM9 (Disintegrin and metalloproteinase domain-containing protein 9)^24^ | A regulator of integrin–matrix interaction |
|  |  | HIF1A (hypoxia inducible factor 1, alpha subunit)^24^ | Inducing angiogenesis |
| **miR-451** |  | Rac-1^25^ | A critical regulator NADPH oxidase and ROS production |
|  |  | MIF (macrophage migration inhibitory factor)^26^ | A proinflammatory cytokine mediating inflammation, monocyte recruitment, and progression of atherosclerosis |
|  |  | COX-2 (cyclooxygenase-2)^27^ | A inflammation mediator |
|  |  | CAB39^28^ | A regulator of PI3K/AKT pathway |

**Reference for Supplementary Table 1.**

1. Lewis BP, Burge CB, Bartel DP. Conserved seed pairing, often flanked by adenosines, indicates that thousands of human genes are microRNA targets. [Cell.](http://www.ncbi.nlm.nih.gov/pubmed/15652477) 2005; 120: 15-20.

2. Li G, Luna C, Qiu J, Epstein DL, Gonzalez P. Alterations in microRNA expression in stress-induced cellular senescence. Mech Ageing Dev. 2009;130:731-741.

3. Petrocca F, Vecchione A, Croce CM. Emerging role of miR-106b-25/miR-17-92 clusters in the control of transforming growth factor beta signaling. Cancer Res. 2008;68:8191-8194.

4. Kim J, Yoon H, Ramírez CM, Lee SM, Hoe HS, Fernández-Hernando C, Kim J.miR-106b impairs cholesterol efflux and increases Aβ levels by repressing ABCA1 expression. Exp Neurol. 2012;235:476-483.

5. Wang H, Liu J, Zong Y, Xu Y, Deng W, Zhu H, Liu Y, Ma C, Huang L, Zhang L, Qin C. miR-106b aberrantly expressed in a double transgenic mouse model for Alzheimer's disease targets TGF-β type II receptor. Brain Res. 2010;1357:166-174.

6. Hua Z, Lv Q, Ye W, Wong CK, Cai G, Gu D, Ji Y, Zhao C, Wang J, Yang BB, Zhang Y. MiRNA-directed regulation of VEGF and other angiogenic factors under hypoxia. PLoS One. 2006;1:e116.

7. Petrocca F, Visone R, Onelli MR, Shah MH, Nicoloso MS, de Martino I, Iliopoulos D, Pilozzi E, Liu CG, Negrini M, Cavazzini L, Volinia S, Alder H, Ruco LP, Baldassarre G, Croce CM, Vecchione A. E2F1-regulated microRNAs impair TGFbeta-dependent cell-cycle arrest and apoptosis in gastric cancer. Cancer Cell. 2008;13:272-286.

8. Carraro G, El-Hashash A, Guidolin D, Tiozzo C, Turcatel G, Young BM, De Langhe SP, Bellusci S, Shi W, Parnigotto PP, Warburton D. miR-17 family of microRNAs controls FGF10-mediated embryonic lung epithelial branching morphogenesis through MAPK14 and STAT3 regulation of E-Cadherin distribution. Dev Biol. 2009;333:238-250.

9. Anton R, Chatterjee SS, Simundza J, Cowin P, Dasgupta R. A systematic screen for micro-RNAs regulating the canonical Wnt pathway. PLoS One. 2011;6:e26257

10. Zhang H, Zuo Z, Lu X, Wang L, Wang H, Zhu Z. MiR-25 regulates apoptosis by targeting Bim in human ovarian cancer. Oncol Rep. 2012;27:594-598.

11. Razumilava N, Bronk SF, Smoot RL, Fingas CD, Werneburg NW, Roberts LR, Mott JL. miR-25 targets TNF-related apoptosis inducing ligand (TRAIL) death receptor-4 and promotes apoptosis resistance in cholangiocarcinoma. Hepatology. 2012;55:465-475.

12. Fang Y, Davies PF. Site-specific microRNA-92a regulation of Kruppel-like factors 4 and 2 in atherosusceptible endothelium. Arterioscler Thromb Vasc Biol. 2012;32:979-987.

13. Li L, Shi JY, Zhu GQ, Shi B.MiR-17-92 cluster regulates cell proliferation and collagen synthesis by targeting TGFB pathway in mouse palatal mesenchymal cells. J Cell Biochem. 2012;113:1235-1244.

14. Tsuchida A, Ohno S, Wu W, Borjigin N, Fujita K, Aoki T, Ueda S, Takanashi M, Kuroda M. miR-92 is a key oncogenic component of the miR-17-92 cluster in colon cancer. Cancer Sci. 2011;102:2264-2271.

15. Taguchi A, Yanagisawa K, Tanaka M, Cao K, Matsuyama Y, Goto H, Takahashi T. Identification of hypoxia-inducible factor-1 alpha as a novel target for miR-17-92 microRNA cluster. Cancer Res. 2008;68:5540-5545.

16. Landais S, Landry S, Legault P, Rassart E. Oncogenic potential of the miR-106-363 cluster and its implication in human T-cell leukemia. Cancer Res. 2007;67:5699-5707.

17. Gabriely G, Wurdinger T, Kesari S, Esau CC, Burchard J, Linsley PS, Krichevsky AM. MicroRNA 21 promotes glioma invasion by targeting matrix metalloproteinase regulators. Mol Cell Biol. 2008;28:5369-5380.

18. Sheedy FJ, Palsson-McDermott E, Hennessy EJ, Martin C, O'Leary JJ, Ruan Q, Johnson DS, Chen Y, O'Neill LA. Negative regulation of TLR4 via targeting of the proinflammatory tumor suppressor PDCD4 by the microRNA miR-21. Nat Immunol. 2010;11:141-147.

19. Chau BN, Xin C, Hartner J, Ren S, Castano AP, Linn G, Li J, Tran PT, Kaimal V, Huang X, Chang AN, Li S, Kalra A, Grafals M, Portilla D, MacKenna DA, Orkin SH, Duffield JS. MicroRNA-21 promotes fibrosis of the kidney by silencing metabolic pathways. Sci Transl Med. 2012;4:121ra18.

20. Ji R, Cheng Y, Yue J, Yang J, Liu X, Chen H, Dean DB, Zhang C. MicroRNA expression signature and antisense-mediated depletion reveal an essential role of MicroRNA in vascular neointimal lesion formation. Circ Res. 2007;100:1579-1588.

21. Han M, Wang Y, Liu M, Bi X, Bao J, Zeng N, Zhu Z, Mo Z, Wu C, Chen X. MiR-21 regulates epithelial-mesenchymal transition phenotype and hypoxia-inducible factor-1α expression in third-sphere forming breast cancer stem cell-like cells. Cancer Sci. 2012;103:1058-1064.

22. Parikh VN, Jin RC, Rabello S, Gulbahce N, White K, Hale A, Cottrill KA, Shaik RS, Waxman AB, Zhang YY, Maron BA, Hartner JC, Fujiwara Y, Orkin SH, Haley KJ, Barabási AL, Loscalzo J, Chan SY. MicroRNA-21 integrates pathogenic signaling to control pulmonary hypertension: results of a network bioinformatics approach. Circulation. 2012;125:1520-1532.

23. Xiong Q, Zhong Q, Zhang J, Yang M, Li C, Zheng P, Bi LJ, Ge F. Identification of novel miR-21 target proteins in multiple myeloma cells by quantitative proteomics. J Proteome Res. 2012;11:2078-2090.

24. Betel D, Wilson M, Gabow A, Marks DS, Sander C. MicroRNA target predictions: The microRNA.org resource: targets and expression. Nucleic Acids Res. 2008; 36(Database Issue): D149-153

25. Wang X, Zhu H, Zhang X, Liu Y, Chen J, Medvedovic M, Li H, Weiss MJ, Ren X, Fan GC. Loss of the miR-144/451 cluster impairs ischaemic preconditioning-mediated cardioprotection by targeting Rac-1. Cardiovasc Res. 2012;94:379-390.

26. Bandres E, Bitarte N, Arias F, Agorreta J, Fortes P, Agirre X, Zarate R, Diaz-Gonzalez JA, Ramirez N, Sola JJ, Jimenez P, Rodriguez J, Garcia-Foncillas J. MicroRNA-451 regulates macrophage migration inhibitory factor production and proliferation of gastrointestinal cancer cells.Clin Cancer Res. 2009;15:2281-2290.

27. Bitarte N, Bandres E, Boni V, Zarate R, Rodriguez J, Gonzalez-Huarriz M, Lopez I, Javier Sola J, Alonso MM, Fortes P, Garcia-Foncillas J. MicroRNA-451 is involved in the self-renewal, tumorigenicity, and chemoresistance of colorectal cancer stem cells.Stem Cells. 2011;29:1661-1671.

28. Tian Y, Nan Y, Han L, Zhang A, Wang G, Jia Z, Hao J, Pu P, Zhong Y, Kang C. MicroRNA-451 downregulates the PI3K/AKT pathway through CAB39 in human glioma.Int J Oncol. 2012;40:1105-1112
