# Supplementary material for: Signature of Circulating MicroRNAs as Potential Biomarkers in Vulnerable Coronary Artery Disease
Source: PLoS One. 2013 Dec 5;8(12):e80738. doi: 10.1371/journal.pone.0080738 (PMC3855151; doi:10.1371/journal.pone.0080738)
Supplement: Protocol S1 — The detailed study design for this observational trial was shown in Protocol S1. (PDF) [file pone.0080738.s002.pdf]

Observational Study Protocol

Title

Screening and identification of circulating microRNAs as potential biomarkers in early diagnosis of vulnerable patients with coronary artery disease

Inclusion Criteria:

- patients with chest pain and suspected coronary artery disease, and with a need to be further confirmed by coronary angiography in the catheterization laboratory at Peking University People's Hospital.

Exclusion Criteria:

- patients with UA caused by other etiological mechanisms (e.g., coronary focal spasm, coronary artery dissection);
- patients with secondary UA related to precipitating factors, such as anemia, fever, tachycardia, hypotension, etc.;
- patients with post-MI UA;
- patients with elevated troponin I (TNI) or creatine kinase (CK-MB) levels;
- patients with a history of severe hepatic and renal dysfunction;
- patients with leukemia, leukopenia or thrombocytopenia;

- patients with ongoing inflammatory and malignant disease.

Start of the study

August , 2012

Scheduled duration

3 years

Expected number of patients

300

SCREENING AND IDENTIFICATION OF CIRCULATING MICRORNAS AS POTENTIAL BIOMARKERS IN EARLY DIAGNOSIS OF VULNERABLE PATIENTS WITH CORONARY ARTERY DISEASE

1. Inclusion/exclusion criteria

Inclusion Criteria:

- patients with chest pain and suspected coronary artery disease, and with a need to be further confirmed by coronary angiography in the catheterization laboratory at Peking University People's Hospital.

Exclusion Criteria:

- patients with UA caused by other etiological mechanisms (e.g., coronary focal spasm, coronary artery dissection);
- patients with secondary UA related to precipitating factors, such as anemia, fever, tachycardia, hypotension, etc.;
- patients with post-MI UA;
- patients with elevated troponin I (TNI) or creatine kinase (CK-MB) levels;
- patients with a history of severe hepatic and renal dysfunction;
- patients with leukemia, leukopenia or thrombocytopenia;

- patients with ongoing inflammatory and malignant disease.

2. Study design

This study intend to include 3 groups of patients that were classified according to angiographic evidence and clinical evaluation of chest pain: (1) control group (n=100); (2) Unstable angina (UA) group (n=100); (3) Stable angina (SA) group (n=100). Patients with chest pain or discomfort but with angiographic exclusion of coronary atherosclerosis were enrolled in the control group. Chest discomfort referred to the following complaints: chest pain, pressure, tightness, or heaviness; pain that radiated to the neck, jaw, shoulders, back, or one or both arms; and persistent shortness of breath. Patients with typical unstable angina (UA) or stable angina (SA), and angiographically documented CAD were enrolled in the UA or SA group. Diagnoses of UA and SA were made according to the ACC/AHA 2007 guidelines for the management of patients with UA/non–ST-elevation MI and the ACC/AHA/ACP-ASIM 1999 guidelines for the management of patients with chronic SA. All UA patients presented with new transient ST-segment abnormalities (≥0.05 mV) that developed during a symptomatic episode and resolved when the symptoms had resolved. There were 3 principal presentations of UA: rest angina (angina commencing when the patient was at rest); new-onset (<2 months) severe angina; and increasing angina (increasing in intensity, duration,

and/or frequency). All patients in the control group presented with normal ECG and had no evidence of ischemia during exercise ECG.

3. Plasma samples collection

EDTA-blood samples (2 mL) were obtained before the cardiac catheterization procedure. Plasma samples will be isolated, frozen (-80℃) and stored to create a large database for measurement of various microRNAs and biological markers analyses, to gain better knowledge on pathophysiology of vulnerable CAD patients. This sampling has been specified in the information for consent.

4. Angiographic analysis

Two experienced interventional cardiologists reviewed all angiographic data to decide the extent of CAD and culprit lesions. When there was a disagreement, the difference was resolved by a further joint analysis. The extent of CAD was defined as one-vessel disease, two-vessel disease, or three-vessel disease according to the number of major coronary vessels with luminal stenosis≥50%.

5. Intravascular Ultrasound (IVUS) Imaging and Analysis*

Intravascular Ultrasound (IVUS) examinations and analyses were performed in accordance with the American College of Cardiology's clinical expert consensus document on IVUS. All IVUS examinations were performed before any intervention and after intracoronary administration of 100 to 200 μg of nitroglycerin using a commercially

available IVUS system (Atlantis SR Pro 2.5F, 40-MHz; Boston Scientific, Natick, Massachusetts, USA). The IVUS catheter was carefully advanced distal to the target lesion, and imaging was performed retrograde back to the aorto-ostial junction with an automatic pullback at 0.5 mm/s. Two independent observers who were blind to the angiographic and clinical findings assessed all IVUS images qualitatively and quantitatively. Discordance with qualitative analysis between the observers was adjusted by assessing together to obtain a consensus. A ruptured plaque was defined as containning a cavity that communicated with the lumen with an overlying residual fibrous cap fragment.

* IVUS was not included in our standard protocol for patient recruitment. We performed this examination only for patients who were willing to receive IVUS and who fit with the appropriate use criteria for IVUS, including patients who were difficult to characterize angiographically (e.g., indeterminate left main stenosis, extent of calcification and dissections) or for sizing of vessel before stent placement.

6. Exercise ECG

Patients with negative angiography results were included in the control group. To rule out UA due to other causes (e.g., microvascular disease, syndrome X, etc.), all of the candidates for the control group

underwent exercise ECG. Only patients with no evidence of ischemia were assessed during follow-up to make the diagnosis. Patients with established non-cardiac disease were eventually enrolled in the control group.

7. Frequency and duration of follow-up

Participants will receive phone calls from Peking University People’s Hospital every three months in 3 years, to make sure they are doing well.
